# Activation of NOD1 and NOD2 in the development of liver injury and cancer

**DOI:** 10.3389/fimmu.2022.1004439

**Published:** 2022-10-04

**Authors:** Naoya Omaru, Tomohiro Watanabe, Ken Kamata, Kosuke Minaga, Masatoshi Kudo

**Affiliations:** Department of Gastroenterology and Hepatology, Kindai University Faculty of Medicine, Osaka-Sayama, Japan

**Keywords:** NOD1, NOD2, hepatocellular carcinoma, microbiota, microbe-associated molecular patterns, pattern recognition receptor

## Abstract

Hepatocytes and liver-resident antigen-presenting cells are exposed to microbe-associated molecular patterns (MAMPs) and microbial metabolites, which reach the liver from the gut *via* the portal vein. MAMPs induce innate immune responses *via* the activation of pattern recognition receptors (PRRs), such as toll-like receptors (TLRs), nucleotide-binding oligomerization domain 1 (NOD1), and NOD2. Such proinflammatory cytokine responses mediated by PRRs likely contribute to the development of chronic liver diseases and hepatocellular carcinoma (HCC), as shown by the fact that activation of TLRs and subsequent production of IL-6 and TNF-α is required for the generation of chronic fibroinflammatory responses and hepatocarcinogenesis. Similar to TLRs, NOD1 and NOD2 recognize MAMPs derived from the intestinal bacteria. The association between the activation of NOD1/NOD2 and chronic liver diseases is poorly understood. Given that NOD1 and NOD2 can regulate proinflammatory cytokine responses mediated by TLRs both positively and negatively, it is likely that sensing of MAMPs by NOD1 and NOD2 affects the development of chronic liver diseases, including HCC. Indeed, recent studies have highlighted the importance of NOD1 and NOD2 activation in chronic liver disorders. Here, we summarize the roles of NOD1 and NOD2 in hepatocarcinogenesis and liver injury.

## Introduction

The liver is exposed to various bacterial components and metabolites derived from the intestinal microbiota *via* the portal vein (1). The anatomical relationship between the liver and gastrointestinal tract creates a unique immunological environment, as the liver needs to maintain immunological tolerance to harmful microbe-associated molecular patterns (MAMPs) of the intestinal microbiota (1, 2). To fulfill this task, the liver contains various types of antigen-presenting cells (APCs), such as Kupffer cells (KCs), dendritic cells (DCs), liver sinusoidal endothelial cells (LSECs), and hepatic stellate cells (HSCs) (2). These unique types of APCs preferentially induce tolerance to food antigens and allografts through the production of anti-inflammatory cytokines and attenuation of responses to toll-like receptor (TLR) ligands (2). Liver APCs with immunosuppressive functions induce tolerance to gut-derived food antigens and MAMPs; however, the presence of these APCs predisposes individuals to viral infections, leading to inflammation-associated hepatocarcinogenesis (2).

Chronic fibroinflammatory disorders of the liver are classified into chronic hepatitis and non-alcoholic steatohepatitis (NASH) (3, 4). The unique immunosuppressive properties of the liver predispose this organ to attack by microorganisms, including hepatitis virus and intestinal microbiota (5). Indeed, the gut-liver axis plays a critical role in the development of chronic liver diseases, especially NASH, as evidenced by the fact that MAMPs and microbial metabolites promote proinflammatory cytokine responses in the liver through the activation of pattern recognition receptors (PRRs) (6). Thus, gut microbiota and hepatitis virus invading the liver cause persistent inflammation due to proinflammatory cytokine responses when MAMPs are sensed by PRRs. Such persistent inflammation also sets the stage for the development of hepatocellular carcinoma (HCC) through inflammation-associated carcinogenesis (7). Most cases of HCC arise from persistent inflammation, e.g., as a result of viral hepatitis or NASH (8, 9). TLRs and nucleotide-binding oligomerization domain (NOD)-like receptors (NLRs) are major PRRs that detect MAMPs derived from the intestinal microbiota (10–12). Liver APCs and hepatocytes express functional TLRs and NOD receptors to detect MAMPs and produce proinflammatory mediators (13, 14). NOD1 and NOD2 are intracellular receptors that recognize muropeptides derived from bacterial cell walls (10). Although the roles of TLRs in the progression of liver injury and cancer are being actively investigated, it remains largely unknown whether activation of NOD1 and NOD2 is beneficial or harmful in these diseases. Given that impaired sensing of intestinal bacteria by NOD1 and NOD2 is associated with several human diseases, including Crohn’s disease and *Helicobacter pylori* infection, it is likely that the progression of liver injury and hepatocarcinogenesis requires activation of NOD1 and NOD2 (10, 11, 15). In this Review, we summarize the recent studies that examined the involvement of NOD1 and NOD2 in hepatocarcinogenesis and liver injury.

## Signaling pathways mediated by NOD1 and NOD2

**Figure 1:**
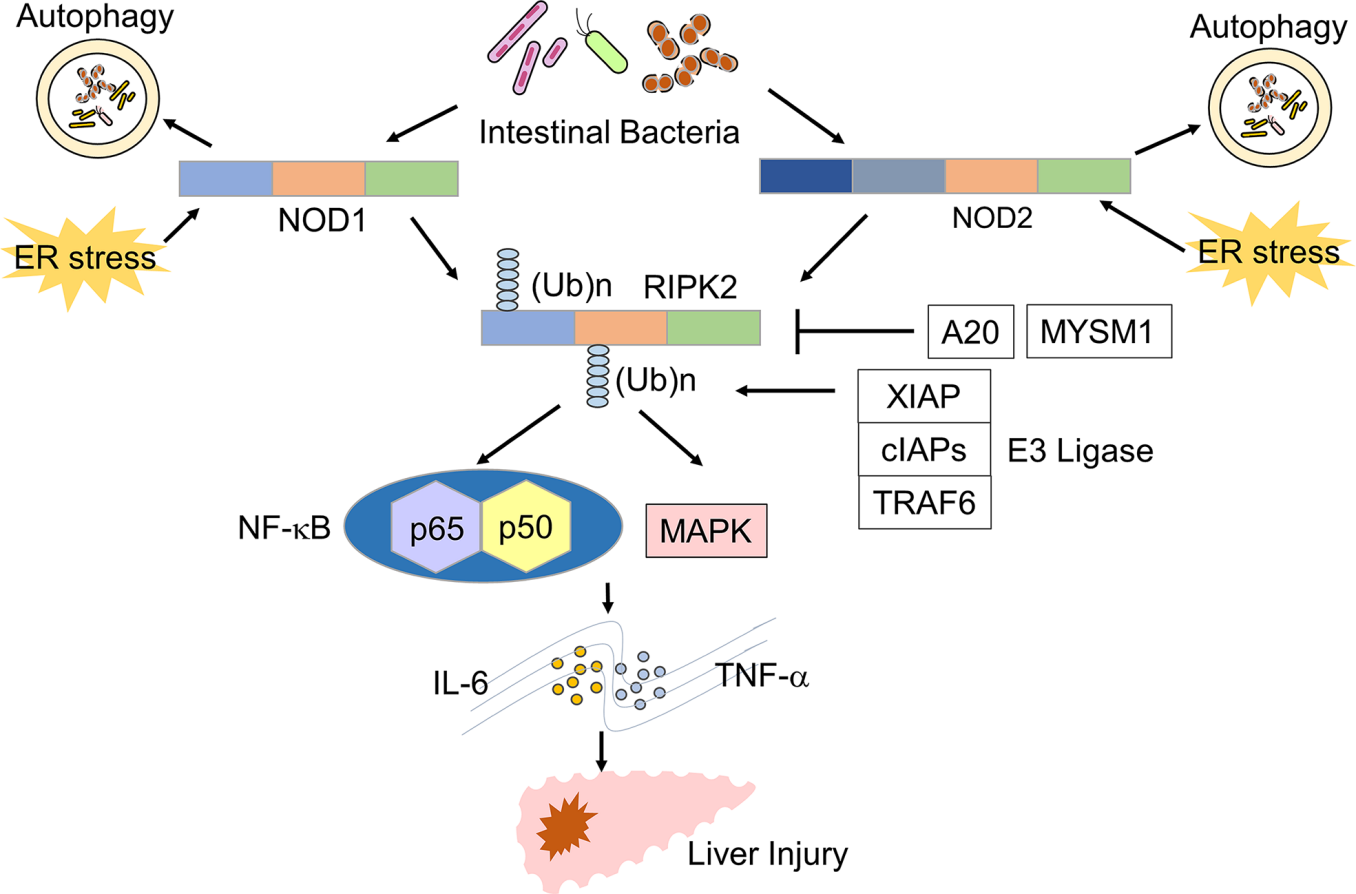
Signaling pathways of NOD1 and NOD2 leading to the development of liver injury. Nucleotide-binding oligomerization domain 1 (NOD1) and NOD2 detect muropeptides derived from the intestinal bacteria and endoplasmic reticulum (ER) stress. Activation of NOD1 and NOD2 leads to the polyubiquitination of receptor-interacting serine/threonine protein kinase 2 (RIPK2). Polyubiquitination of RIPK2 requires molecular interactions between RIPK2 and E3 ligases, including X-linked inhibitor of apoptosis (XIAP), cellular inhibitor of apoptosis proteins (cIAPs), and TNF-receptor associated factor 6 (TRAF6). A20 and MYSM1 remove polyubiquitin chains from RIPK2. Activation of RIPK2 induces the production of TNF-α and IL-6 through the nuclear translocation of NF-κB subunits and activation of the mitogen-activated protein kinase (MAPK) pathway and thereby promotes the development of liver injury.

**Figure 2:**
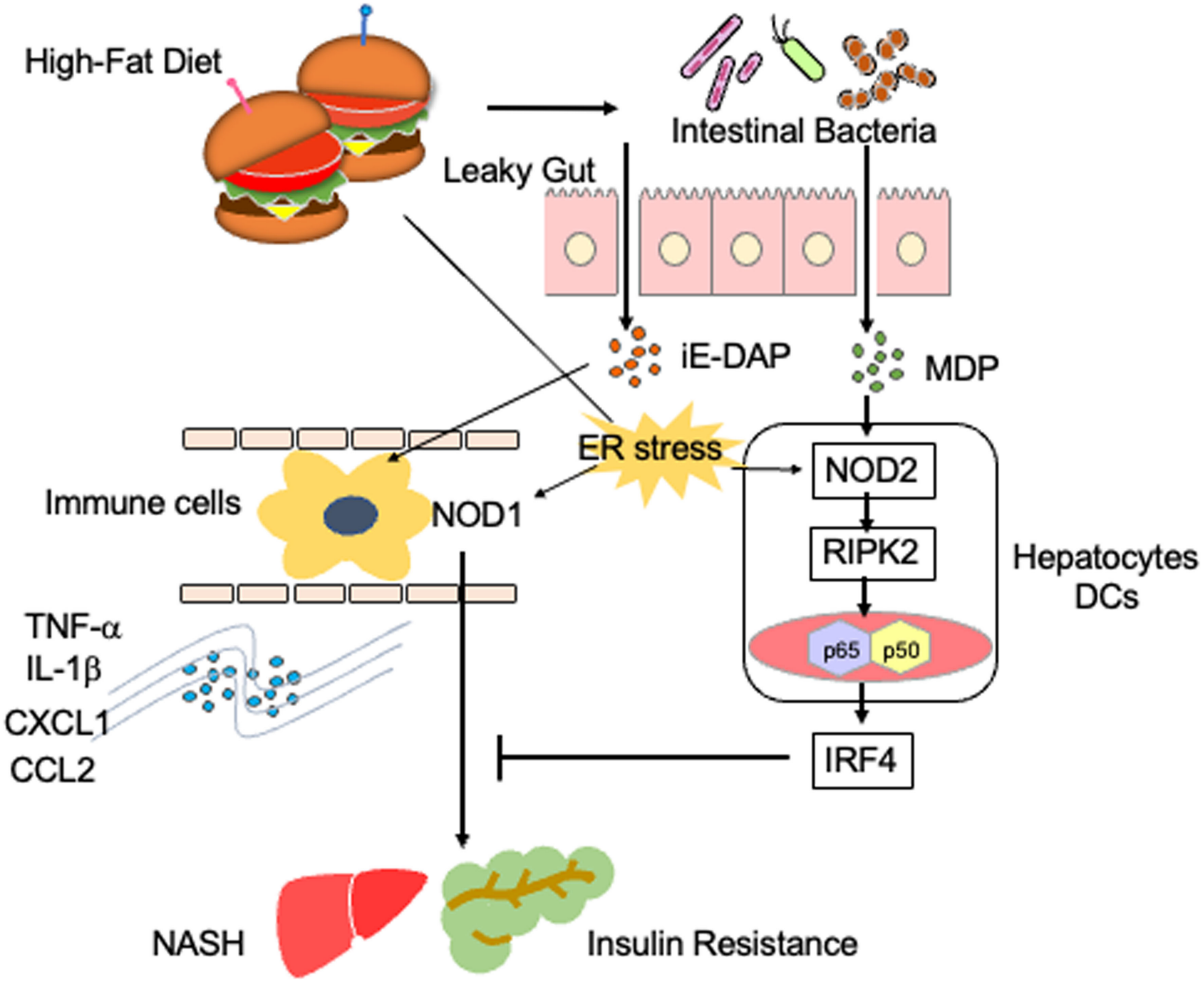
Proinflammatory roles played by NOD1 and NOD2 in steatosis. A high-fat diet causes gut leakage and activates NOD1 and NOD2 in gastrointestinal organs. γ-D-glutamyl meso-diaminopimelic acid (iE-DAP) derived from intestinal bacteria activates NOD1 in circulating immune cells. NOD1 activation in circulating immune cells results in the development of NASH and insulin resistance *via* the production of IL-1β and TNF-α. In contrast, muramyl dipeptide (MDP) derived from intestinal bacteria activates NOD2, which is expressed in hepatocytes or dendritic cells (DCs). The activation of NOD2 by MDP induces the expression of IFN regulatory factor 4 (IRF4) and thereby suppresses NOD1-mediated proinflammatory cytokine responses. Endoplasmic reticulum (ER) stress also activates NOD1 and NOD2.

NOD1 and NOD2 are expressed in innate immune cells, such as macrophages, DCs, KCs, LSECs, and hepatocytes (10, 14, 16, 17). NOD1 and NOD2 are intracellular receptors for muropeptides derived from bacterial cell wall components, such as peptidoglycan (PGN) (10, 16). Tripeptide-A-γ-D-glutamyl meso-diaminopimelic acid (Tripeptide-A-iE-DAP) and muramyl dipeptide (MDP) are the minimal motifs recognized by NOD1 and NOD2. Thus, these molecules are widely used as NOD1 and NOD2 ligands (**Figures 1**, **2**) (10, 16). The main outcome of the stimulation of NOD1 and NOD2 is the activation of transcription factors, including nuclear factor-κB (NF-κB), interferon regulatory factor 3 (IRF3), and IRF7 (10, 15, 16). In addition to nuclear translocation of NF-κB and IRFs, sensing of bacterial components by NOD1 and NOD2 leads to activation of mitogen-activated protein kinases (MAPKs) through TGF-β-activated kinase 1 (TAK1) (10, 15, 16). The activation of NF-κB and IRFs by NOD1 and NOD2 depends upon the molecular interaction between NOD receptors and receptor interacting serine/threonine protein kinase 2 (RIPK2) (**Figure 1**) (10, 16). NF-κB activation caused by the stimulation of NOD1 and NOD2 results in the release of proinflammatory cytokines and chemokines, such as IL-6, TNF-α, and C-C motif chemokine ligand 2 (CCL2), whereas nuclear translocation of IRF3 and IRF7 leads to the production of type I interferons (IFNs) (10, 15, 16). Thus, activation of NOD1 and NOD2 induced by the recognition of components derived from intestinal bacteria results in proinflammatory and type I IFN responses.

RIPK2 is a downstream signaling molecule activated by NOD1 and NOD2, and its activation is tightly regulated by polyubiquitination (18) (**Figure 1**). Lys (K)63-linked polyubiquitination of RIPK2 is necessary for NF-κB activation. E3 ligases, including cellular inhibitor of apoptosis 1 (cIAP1), cIAP2, Pellino3, TNF-receptor factor 6 (TRAF6), and X-linked inhibitor of apoptosis protein (XIAP), mediate K63-linked polyubiquitination (18). In addition to K63-linked polyubiquitination, RIPK2 undergoes N-terminal methionine (M1)-linked polyubiquitination mediated by the linear ubiquitination chain assembly complex (LUBAC) when RIPK2 interacts with XIAP (18). K63-and/or Met1-linked polyubiquitination modifications are necessary for the nuclear translocation of NF-κB subunits following RIPK2 activation. Polyubiquitination of RIPK2 activates a downstream signaling cascade involving TAK1 and IκB kinase (IKK) complex composed of IKKα, IKKβ, and IKKγ (10, 18). Negative regulators of NF-κB activation suppress RIPK2 polyubiquitination. For example, IRF4 and autophagy-related 16 like 1 (ATG16L1) activated upon sensing of MDP by NOD2, inhibit K63-linked polyubiquitination of RIPK2, thereby suppressing proinflammatory cytokine responses (19–21). In addition, Myb like, SWIRM and MPN domains 1 (MYSM1) and A20 have been shown to dampen NF-κB activation by RIPK2 through the removal of the K63 and M1-linked polyubiquitin chains (22, 23).

Although NOD2 activation causes activation of NF-κB and type I IFN pathways in a RIPK2-dependent manner, sensing of cytosolic single-stranded RNA by NOD2 induces type I IFN production *via* mitochondrial antiviral signaling protein-dependent and RIPK2-independent mechanisms (24). In addition, sensing of PGN by NOD1 and/or NOD2 leads to the molecular interaction between ATG16L1 and NOD1/NOD2 and thereby induces autophagy without involvement of RIPK2 activation (25).

Activators for NOD1 and NOD2 are not limited to the fragments of bacterial PGN. Recent studies provide evidence that NOD1 and NOD2 function as cytosolic and endoplasmic reticulum (ER) stress sensors (26–29). Endogenous metabolite sphingosine-1-phosphate induces NF-κB activation through direct binding to NOD1 and NOD2 (30). ER-stress inducers, thapsigargin and dithiothreitol, promotes IL-6 production in a NOD1/NOD2-depdendent manner through activation of TRAF2 (29). These new studies support the notion that NOD1 and NOD2 function as sensors not only for microbial components but also for endogenous danger signals to promote and suppress inflammation.

## Activation of NOD1 and NOD2 in liver injury

Liver-resident APCs such as DCs, KCs, and LSECs express functional NOD1 and NOD2 (14, 17, 31). NOD2-deficient mice were resistant to the induction of autoimmune hepatitis (AIH) induced by concanavalin A (ConA) compared to the susceptibility of NOD2-intact mice, and this resistance was associated with reduced expression of IFN-γ-in the liver (32). Consistent with this, MDP activation of NOD2 acted synergistically with ConA to induce severe AIH (32). Such synergistic action of MDP and ConA on the development of AIH was accompanied by the expression of IFN-γ and TNF-α (32). Given that hepatocytes and APCs constitutively express NOD2, these results suggest that NOD2 mediates the development of AIH through pro-inflammatory cytokine responses.

Injection of D-galactosamine (D-Gal) in combination with lipopolysaccharide (LPS) is widely used to induce acute liver failure (ALF) (33, 34). Recent studies have highlighted the importance of RIPK2 polyubiquitination in this model. Damagaard et al. reported that pretreatment with MDP increased the severity of ALF induced by D-Gal and LPS through the increase of proinflammatory cytokines such as IL-6 and TNF-α (34). Mice deficient in XIAP, an E3 ligase mediating RIPK2 polyubiquitination, displayed attenuated ALF induced by MDP pre-sensitization in this D-Gal/LPS model, suggesting that polyubiquitination of RIPK2 by XIAP is required for the development of severe ALF (34). Furthermore, *in vitro* studies showed that recruitment of LUBAC to RIPK2 as well as K63-likned polyubiquitination are necessary for the optimal NF-κB activation and subsequent production of IL-6 and TNF-α (34). These results suggest that the NOD2-mediated activation of RIPK2 by K63-linked polyubiquitination and LUBAC recruitment plays a pathogenic role in the development of severe ALF (**Figure 1**). Conversely, Panda et al. provided evidence that mice deficient in MYSM1, a deubiquitinase of RIPK2, have increased levels of IL-6, TNF-α, and serum liver enzymes upon MDP injection compared with those in mice with intact MYSM1 (23). Thus, MYSM1 attenuates NOD2-mediated liver injury by removing polyubiquitination of RIPK2. These recent studies strongly suggest that activation of NOD2 mediates liver injury through RIPK2 polyubiquitination.

In contrast to the sensitizing action of NOD2, pre-activation of NOD1 by the NOD1 ligand C14-Tri-LAN-Gly markedly inhibited the development of ALF induced by D-Gal/LPS (33). Suppression of ALF by NOD1 activation was associated with enhanced expression of A20 in hepatocytes (33). Given that A20 removes polyubiquitin chains from RIPK2, it is likely that NOD1 suppresses ALF through RIPK2 deubiquitination (22). On the other hand, NOD1 contributed to the development of acute liver injury caused by the exposure to carbon tetrachloride (CCl_4_) (35). NOD1-deficent mice were protected from the CCl_4_-induced acute liver injury, and this resistance was accompanied by reduced migration of neutrophils into the liver (35). Such discrepancies in the effects of NOD1 on liver pathologies induced by D-Gal/LPS and CCl_4_ could be partially explained by differences in the types of immune cells recruited to the liver: the latter model is driven by hepatic infiltration of neutrophils rather than lymphocytes (35).

TLRs are the major PRRs for the detection of MAMPs (12). Myeloid differentiation factor 88 (MyD88) is a downstream signaling molecule for TLRs (12). MyD88-deficient mice were protected from liver damage induced by ConA owing to the downregulation of TNF-α, IL-6, and IFN-γ expression levels (36). Thus, TLR-MyD88 signaling pathways are involved in the development of AIH. We and others showed that NOD2 negatively regulates TLR-mediated proinflammatory cytokine responses (19, 20, 37, 38). As for the molecular mechanisms accounting for the downregulation of TLR-mediated signaling pathways, activation of NOD2 by MDP in DCs leads to the expression of IRF4, which inhibits TLR-mediated signaling pathways by binding to MyD88, TRAF6, and RIPK2 (19, 20, 37, 38). Therefore, it is possible that the development of TLR-dependent liver injury is suppressed by IRF4 induced by the activation of NOD2 with MDP. Indeed, proinflammatory cytokine production induced by the TLR9 ligand CpG in liver plasmacytoid DCs was markedly reduced upon the stimulation with MDP, and accompanied by the induction of IRF4 expression (39). Although no reports have addressed whether NOD2 inhibits the development of liver injury induced by TLRs, the TLR-dependent liver damage may be successfully treated by the activation of NOD2 with MDP.

## Activation of NOD1 and NOD2 in steatosis

Activation of NOD1 and NOD2 is involved in the development of metabolic syndromes (26). In animal models, NOD1 has been shown to contribute to the development of insulin resistance and metabolic syndromes caused by the high-fat diet (HFD) (40). Schertzer et al. reported that mice deficient in both NOD1 and NOD2 were protected from hepatic lipid accumulation caused by the HFD (41). Injection of a NOD1 ligand into mice led to adipose tissue inflammation and insulin resistance (41). Moreover, administration of gefitinib, a RIPK2 inhibitor, attenuated metabolic inflammation and insulin resistance caused by NOD1 activation (42). Expression of NOD1 in hematopoietic cells has been highlighted as a molecular mechanism accounting for the development of metabolic inflammation and insulin resistance (**Figure 2**) (43). Enhanced intestinal leakiness induced by the HFD leads to the accumulation of NOD1 ligands in the serum as a result of increased bacterial translocation (43). NOD1 expressed in circulating hematopoietic cells recognizes NOD1 ligands and induces the production of C-X-C motif chemokine ligand 1 (CXCL1) by macrophages to attract neutrophils into the adipose tissues (43). In line with these findings, HFD-fed mice displayed progressive impairment of insulin signaling, as was evidenced by the impaired activation of AKT in the skeletal muscle (44). Impairment of insulin signaling paralleled the increase in intestinal permeability and accumulation of NOD1 ligands derived from the intestinal bacteria in the serum (44). Thus, NOD1 not only functions as a PRR for intestinal bacterial components but also stimulates the development of insulin resistance and metabolic syndrome, including steatosis.

NOD1-mediated insulin resistance and obesity are negatively regulated by IRF4, which is induced by the activation of NOD2 with MDP (37). Injection of MDP into HFD-fed mice markedly reduced the expression of proinflammatory cytokines and chemokines, such as TNF-α, IL-1β, CXCL1, CXCL9, CXCL10, and CCL2, in white adipose tissue (37). This suppressive effect of MDP on the proinflammatory cytokine and chemokine responses was not seen in mice deficient in IRF4 (37). Thus, NOD1 and NOD2 play, respectively, pathogenic and protective roles in the development of metabolic inflammation (37). In line with this idea, NOD2-deficient mice maintained on the HFD displayed enhanced metabolic inflammation (45, 46). Higher accumulation of T cells and myeloid cells producing IL-6 and TNF-α was observed in the livers of HFD-fed NOD2-deficient mice compared to that in the livers of NOD2-intact mice (45, 46). NOD2 expressed in non-hematopoietic cells, rather than in hematopoietic cells, protects against insulin resistance and metabolic inflammation, because hepatocyte-specific NOD2 deletion resulted in the development of severe steatosis and hepatic fibrosis (47). Indeed, expression levels of the T helper type 1 (Th1) chemokine CXCL9 and profibrogenic cytokine TGF-β1 was enhanced in mice with hepatocyte-specific NOD2 deficiency (47). Furthermore, a bone marrow transplantation experiment revealed that non-hematopoietic expression of RIPK2 is required for the NOD2-mediated protection against insulin resistance and metabolic syndrome (48).

As mentioned earlier, increased metabolic inflammation and insulin resistance are associated with the translocation of gut microbiota into adipose tissue and the liver due to the impaired intestinal barrier. Metabolic inflammation and insulin resistance are driven by the sensing of translocated intestinal microbiota by NOD1, which is downregulated by the activation of NOD2. The ER stress is a major trigger for the development of insulin resistance and obesity (49), and it has been shown to activate NOD1 and NOD2 (26). Therefore, it is possible that insulin resistance and metabolic inflammation can be regulated by the activation of NOD1 and NOD2 through the recognition of intestinal bacteria or *via* the stimulation by the ER stress.

## Involvement of NOD1 and NOD2 in hepatocarcinogenesis

Bacterial components and metabolites carried to the liver from the gastrointestinal tract *via* the portal vein include MDP (a NOD2 ligand), iE-DAP (a NOD1 ligand), lipoteichoic acid (LTA, a TLR2 ligand), LPS (a TLR4 ligand), deoxycholic acid (DCA), and short-chain fatty acids (SCFA) (50). Thus, immune responses caused by these microbial components and metabolites are involved in hepatocarcinogenesis (50). Persistent inflammation plays an important role in the development of HCC, as demonstrated by the established notion that hepatitis virus and metabolic syndromes, which cause chronic liver injury, are strong risk factors for hepatocarcinogenesis (7, 9). Chronic liver injury, which leads to compensatory liver regeneration, fibrosis, and cirrhosis, is observed in many cases of HCC (9). A single administration of the carcinogen, diethylnitrosamine (DEN) in combination with repeated injections of CCl_4,_ has been widely used to create an experimental model of HCC. In this model, repeated liver injuries induced by CCl_4_ are exacerbated by DNA damage induced by DEN to mimic inflammation-associated hepatocarcinogenesis. Recent data obtained from the DEN/CCl_4_ HCC model supports the view that MAMPs and microbial metabolites entering the liver can be possible triggers of hepatocarcinogenesis.

The pathogenic role of NOD2 in the development of liver injury prompted researchers to examine the involvement of this PRR in hepatocarcinogenesis. Zhou et al. showed that NOD2 promotes hepatocarcinogenesis through proinflammatory cytokines and autophagic responses *via* RIPK2 activation (51). They found that expression levels of NOD2 and phosphorylated RIPK2 were higher in human HCC tissues than in noncancerous tissues (51). Based on the results of human studies, they also examined whether NOD2 promotes hepatocarcinogenesis in the DEN/CCl_4_ model and found that HCC and inflammation were significantly attenuated in mice with hepatocyte-specific NOD2 or RIPK2 knock-out mice (51). Hepatocyte-specific NOD2 or RIPK2 deletion led to decreased activation of two oncogenic transcription factors, signal transducer and activator of transcription 3 (Stat3) and NF-κB, which resulted in diminished expression of proinflammatory cytokines such as IL-6 and TNF-α. Thus, NOD2 activation promotes inflammation-associated hepatocarcinogenesis in a RIPK2-dependent manner.

Obesity and NASH promote the development of HCC (7). Combined treatment with the carcinogen dimethylbenzanthracene (DMBA) and HFD is widely used as an experimental model of NASH-associated HCC (52). DCA is a secondary bile acid synthesized from the primary bile acids by intestinal bacteria. DNA damage may be induced in the liver exposed to DCA (52). Yoshimoto et al. addressed the role of DCA in the development of obesity-associated HCC in this model (52). DCA activates HSCs, which acquire the senescence-associated secretory phenotype (SASP) and produce IL-1β, IL-6, CXCL1, and CXCL9, thereby facilitating the emergence of the tumor microenvironment (52).

Given that NOD2 protects mice from HFD-induced NASH, Gurses et al. investigated whether NOD2-deficient mice are sensitive to NASH-associated liver cancer and showed that upon the treatment with DMBA and consumption of HFD, NOD2-deficient mice gained more weight and bore more HCC tumors than NOD2-intact mice (45). Enhanced activation of Stat3 and infiltration of immune cells were associated with increased hepatocarcinogenesis in NOD2-deficient mice treated with DMBA and HFD (45). Although HSCs with SASP play pivotal roles in obesity-dependent hepatocarcinogenesis (50), the effects of NOD2 activation on HSCs have not yet been explored. In line with the data obtained in obesity-associated HCC, Ma et al. examined the involvement of NOD2 in the model of HCC induced by a combined treatment with DEN and CCl_4_ (53). They found that NOD2 acted as a tumor suppressor, as more HCC tumors were seen in the liver of NOD2-deficient mice than in the liver of NOD2-intact mice (53). Expression of NOD2 was significantly decreased in human liver regions affected by HCC compared to that in the non-cancerous tissue (53). In addition, *in vitro* studies in human HCC cell lines revealed that NOD2 is required to enhance sensitivity to sorafenib and lenvatinib through the activation of the adenosine 5′-monophosphate-activated protein kinase (AMPK) pathway (53). As for molecular mechanism, accounting for the NOD2-mediated inhibition of HCC growth, NOD2 induces autophagy-mediated apoptosis of HCC through its interaction with AMPK-α and LKB1. In these models of experimental hepatocarcinogenesis, NOD2 acted not only as a tumor suppressor but also as a chemotherapy enhancer (45, 53).

As mentioned above, data regarding the sensitivity to carcinogen-induced hepatocarcinogenesis in NOD2-deficient mice have been conflicting (51, 53). The reasons why NOD2 has oncogenic activity in the DEN/CCl_4_ model remain unknown at present. Differences in cell types expressing NOD2 may explain this discrepancy. In the DEN/CCl_4_ model, Zhou et al. observed fewer HCC tumors in mice with hepatocyte-specific NOD2 or RIPK2 deficiency, whereas in mice with NOD2 knockout in both hepatocytes and hematopoietic cells, the number of HCC tumors was increased (51, 53). Therefore, it is possible that NOD2 activation in KCs and DCs protects mice from hepatocarcinogenesis and metabolic syndrome, whereas NOD2 activation in hepatocytes promotes oncogenesis (51). Confirmation of this idea awaits the results of experiments in which mice with NOD2 deficiency specifically in myeloid cells are challenged with DEN/CCl_4_. Contrasting data on hepatocarcinogenesis by NOD2 activation may be not surprising. NOD2 activation has been shown to suppress anti-cancer immunity induced by the gut colonization with *Enterococcus hirae* and *Barnesiella intestinihominis* (54, 55). On the other hand, PGN sensing by NOD2 can be deleterious in the intestinal epithelium (54, 55). Given such multifaceted roles by NOD2, it is possible that NOD2 may both positively and negatively regulate the hepatocarcinogenesis.

**Figure 3:**
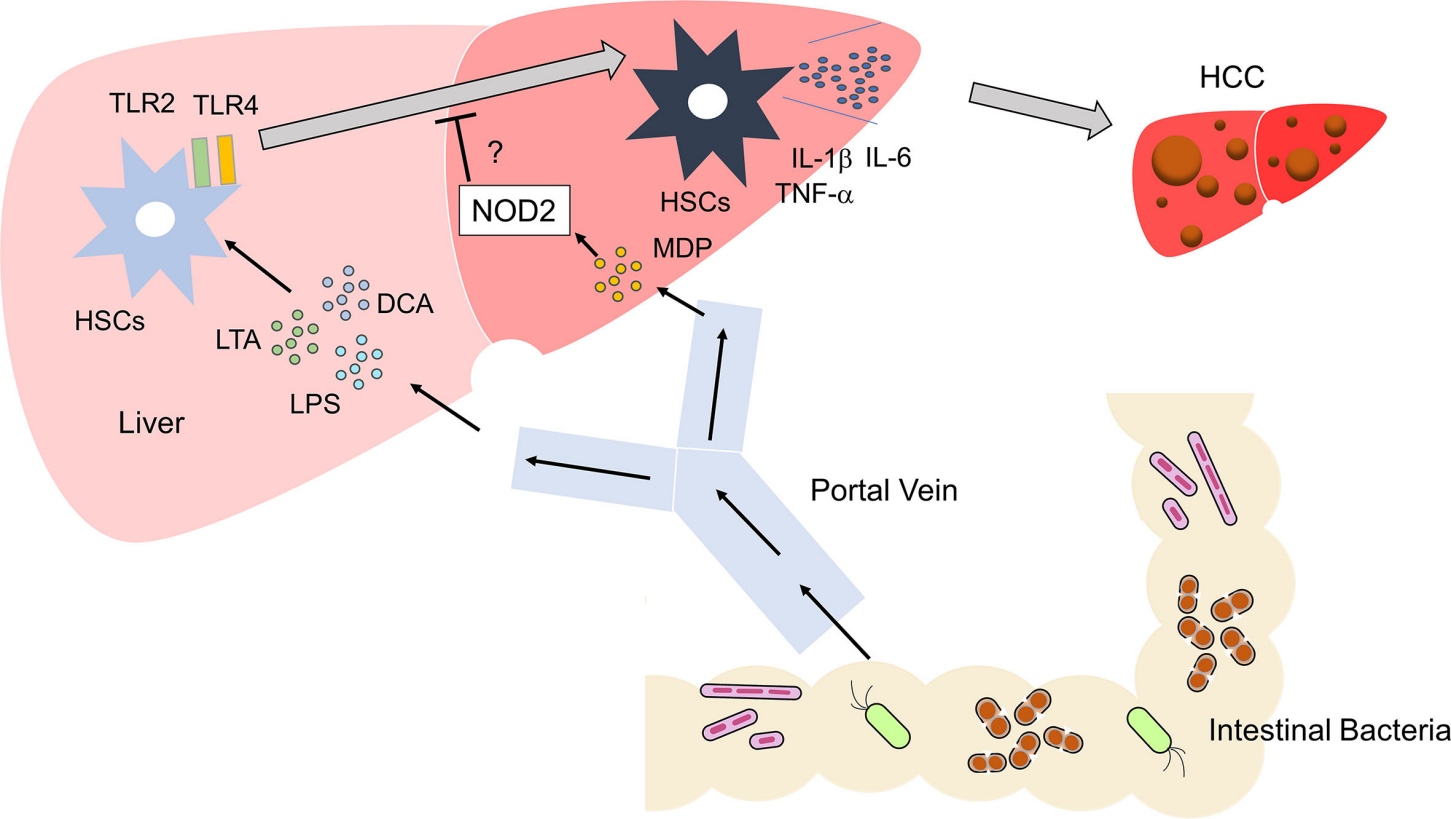
Activation of TLRs and NOD2 in the development of liver cancer Microbe-associated molecular patterns (MAMPs), including lipopolysaccharide (LPS) and lipoteichoic acid (LTA), enter the liver *via* the portal vein. These MAMPs activate TLR2 and TLR4 in hepatic satellite cells (HSCs). Metabolites such as deoxycholic acid (DCA) act together with TLR2 and TLR4 ligands to induce differentiation of HSCs with a senescence-associated secretory phenotype (SASP). HSCs with SASP produce large amounts of IL-1β, IL-6, and TNF-α. Differentiation of regular HSCs into those with SASP promotes the development of HCC. Activation of NOD2 by MDP might inhibit the development of HCC through its negative regulation of TLR2 and TLR4.

If activation of NOD2 negatively regulates TLR-mediated chronic inflammation, it is likely that NOD2 attenuates inflammation-associated cancer driven by TLRs (**Figure 3**). In fact, activation of NOD2 by MDP suppressed colorectal tumorigenesis through IRF4-mediated inhibition of TLR signaling pathways (38). This scenario might also apply to the development of HCC. Recognition of the intestinal microbiota by TLR4 is required to trigger the development of HCC in the DEN/CCl_4_ model (56, 57). In another inflammation-associated HCC model, the occurrence of NASH-associated HCC was markedly decreased in TLR2-deificinet mice (50). Thus, activation of TLRs is an indispensable step in hepatocarcinogenesis. TLR-mediated activation of NF-κB and production of proinflammatory cytokines were markedly suppressed in the colonic mucosa of experimental murine colitis upon the activation of NOD2 by MDP (19, 20), which may also protect mice from hepatocarcinogenesis induced by NASH or treatment with DEN/CCl_4_. However, to the best of our knowledge, the mechanisms suppressing hepatocarcinogenesis have not been examined with respect to the crosstalk between NOD2 and TLRs.

Although several reports have addressed the role of NOD2 in experimental models of HCC, no study has examined the role of NOD1. However, the effects of NOD1 on cell survival and proliferation have been tested *in vitro* (58). Expression of NOD1 was found to be significantly lower in the HCC tissue than in the non-cancerous parts of the liver (58). NOD1 activation suppressed HCC proliferation through the inhibition of SRC and induction of cell cycle arrest at the G1 phase (58). In addition, overexpression of NOD1 in HepG2 and Huh7 cells resulted in higher sensitivity to sorafenib (58). Despite *in vitro* data alone, these data suggest that NOD1 can suppress the growth of HCC *via* the downregulation of SRC activity and cell cycle progression.

## Conclusion

The activation of NOD1 and NOD2 is involved in the development of liver injury and hepatocarcinogenesis. Conflicting data have been reported: NOD2 activation is required for liver injury, whereas NOD1 activation plays both protective and pathogenic roles in the development of hepatitis. Similarly, administration of NOD1 and NOD2 ligands exacerbated and improved steatosis, respectively. The NOD2 signaling pathways are both beneficial and pathogenic in hepatocarcinogenesis. Further elucidation of the molecular mechanisms by which NOD1 and NOD2 activation regulate the development of liver injury and cancer is required for the application of NOD1 and NOD2 ligands as treatments of human diseases. Immune checkpoint inhibitors (ICIs) targeting programmed death-1 (PD-1) or cytotoxic T lymphocyte antigen-4 (CTLA-4) are widely used to treat advanced solid cancers, including HCC (9). However, the efficacy of ICIs alone for HCC is 20%, as determined by the response rate (9). Therefore, the restoration of anti-cancer Th1 immunity by ICIs alone is not sufficient. A novel combination immunotherapy consisting of ICIs and compounds that enhance T cell immunity needs to be established. In this regard, ligands for NOD1 or NOD2 can be promising candidates, because activation of NOD1 and NOD2 efficiently induces Th1 responses (15, 59). In addition to conventional PGN sensors, recent studies highlight roles played by the NOD1 and NOD2 in the maintenance of ER homeostasis. Molecular mechanisms accounting for the development of liver injury and HCC through regulation of autophagy and ER stress by NOD1 and NOD2 need to be addressed in the future studies. In conclusion, the elucidation of the association between NOD1/NOD2-mediated signaling pathways and liver diseases opens new avenues for the development of novel treatments for hepatitis, steatosis, and HCC.

## Author contributions

NO and TW drafted the manuscript and prepared figures. MK and KK reviewed the manuscript for intellectual content. KM, TW, and NO were responsible for the revision of the manuscript. All authors contributed to the article and approved the submitted version.

## Funding

This work was supported by Grants-in-Aid for Scientific Research (22K07996, 22K08090, 21K15987) from the Japan Society for the Promotion of Science, Takeda Science Foundation, Yakult Bio-Science Foundation, SENSHIN Medical Research Foundation, Smoking Research Foundation and a 2022 Kindai University Research Enhancement Grant (KD2208).

## Acknowledgments

We thank Ms. Yukiko Ueno for her secretarial assistance.

## Conflict of interest

The authors declare that the research was conducted in the absence of any commercial or financial relationships that could be construed as a potential conflict of interest.

## Publisher’s note

All claims expressed in this article are solely those of the authors and do not necessarily represent those of their affiliated organizations, or those of the publisher, the editors and the reviewers. Any product that may be evaluated in this article, or claim that may be made by its manufacturer, is not guaranteed or endorsed by the publisher.
